# Corona−Poled Porous Electrospun Films of Gram−Scale Y−Doped ZnO and PVDF Composites for Piezoelectric Nanogenerators

**DOI:** 10.3390/polym14183912

**Published:** 2022-09-19

**Authors:** Juan Yi, Yiheng Song, Shixian Zhang, Zhilong Cao, Chenjian Li, Chuanxi Xiong

**Affiliations:** School of Materials Science and Engineering, Wuhan University of Technology, Wuhan 430070, China

**Keywords:** poly(vinylidene fluoride), Y−ZnO, electrospining, corona poling, wearable device

## Abstract

For digging out eco−friendly and well−performed energy harvesters, piezoelectric nanogenerators are preferred owing to their effortless assembly. Corona−poling promotes output performance of either aligned or porous PVDF electrospun films and higher piezoelectric output was achieved by corona−poled porous PVDF electrospun films due to more poled electret dipoles in pores. Increasing the duration of electrospinning rendered more electret dipoles in PVDF porous electrospun films, resulting in higher piezoelectric output. Moreover, corona−poled PVDF/Y−ZnO porous electrospun films performed better than corona−poled PVDF/ZnO porous electrospun films because of the larger polar crystal face of Y−ZnO. Flexible piezoelectric polymer PVDF and high−piezoelectric Y−ZnO complement each other in electrospun films. With 15 wt% of Y−ZnO, corona−poled PVDF/Y−ZnO porous electrospun films generated maximum power density of 3.6 μW/cm^2^, which is 18 times that of PVDF/BiCl_3_ electrospun films.

## 1. Introduction

Green energy harvesters [1] are in urgent need because of sustainability [2] and excessive dependence of non−renewable energy, such as petroleum [3]. Piezoelectric nanogenerators (PENGs) transcend triboelectric nanogenerators owing to better resistance of disturbance from surrounding static electricity [4]. General PENGs are fabricated by a high−piezoelectric filler and a flexible polymer [5]. Considering the toxicity of Pb, BaTiO_3_ thrived in area of PENGs with satisfactory piezoelectricity. A high temperature during sintering obstructed its further development, thus eco−friendly but low−piezoelectric ZnO−based fillers are emphasized. Subsequently, the core problem is how to enhance the piezoelectricity of a ZnO−based filler. With Fe^3+^ dopant in ZnO, a high piezoelectric coefficient (*d*_33_) of 110 pC/N can be obtained since the ionic size of Fe^3+^ (0.64 Å) is much smaller than that of Zn^2+^ (0.74 Å) and Fe−O bond is easier to rotate [6]. With Ca^2+^ dopant in ZnO, the bandgap energy of Ca−doped ZnO (3.20 eV) was smaller than that of ZnO (3.24 eV), leading to an output voltage of 3 V in PVDF−TrFE/ Ca−doped ZnO film with finger tapping [7].

Among ZnO−based piezoelectric ceramics, Y−doped ZnO (Y−ZnO) own high *d*_33_* of ~420 pm/V. When Y−ZnO was blended with PDMS, output voltage density of approximately 3.3 V/cm^2^ can be obtained [8]. However, the issue of material recycle will hinder the sustainability of PDMS/Y−doped ZnO PENGs due to thermosetting of PDMS, and non−piezoactive PDMS impeding the enhancement of piezoelectricity. In our previous work, PVDF/Y−ZnO aligned electrospun films were fabricated due to the merit of dissolvability of electrospun films in DMF, and increasing dipole alignment was verified as mechanism of enhanced piezoelectricity [9]. Additionally, electrospinning duration can affect the thickness of electrospun films, and a thicker film can generate higher piezoelectric output [10]. As for the speed of the rotating collector, a collector that was too fast (>2000 rpm) made electrospun fibers aligned on almost one direction of rotating but impeded heat dissipation during electrospinning, thus the electrospinning process was divided into several segments with intermission [11]. A slow collector (<100 rpm) has the edge on lower heat production, guaranteeing long running capability for consecutively manufacturing porous electrospun films [12].

For enhancing piezoelectricity in porous electrospun films, direct poling is arduous to execute with a possible short circuit when coating electrodes on electrospun films, while corona poling without electrodes is effective and vital for poling airs in pores [13]. A below air breakdown electric field of 30 kV/cm, corona between needle and bottom metal plate, signifies that air in pores was ionized but not broken down without destruction and failure. After ionization, air in porous electrospun films can be ionized as pairs of positive and negative charge, which lead to increasing electret dipoles and an increasing piezoelectric response [14].

In this work, elongated electrospun duration was used for improving piezoelectric output. Next, corona poling was utilized for both aligned and porous PVDF electrospun films, exploring piezoelectric output with more electret dipoles. Then, Y−doped ZnO and PVDF porous electrospun films were fabricated for probing piezoelectricity with an appropriate content of piezoelectric filler. At last, PVDF/Y−ZnO porous electrospun films can be used for harvesting human movements, either walking or running.

## 2. Experimental Section

### 2.1. Materials

Zinc nitrate hexahydrate (AR grade), sodium hydroxide (AR grade), and N,N−dimethylformamide (AR grade) were purchased from Sinopharm Chemical Reagent Co., Ltd. (Shanghai China). PVDF powder (Solef 6010) was achieved from Solvay (China) Co., Ltd. (−Shanghai, China). Yttrium chloride (99.95% metals basis) was obtained from Shanghai Aladdin Reagent Co., Ltd. (Shanghai, China). Deionized water was obtained in our lab.

### 2.2. Synthesis of Y−ZnO and ZnO

A total of 15 g Zn (NO_3_)_2_⋅6H_2_O was dissolved in 50 mL deionized water, and 5 mol% YCl_3_ was mixed in the solution. Next, the solution was instilled into an NaOH aqueous solution with stirring and these chemicals reacted overnight. Then, white precipitates were obtained by centrifugation and rinsing with water. Finally, dried white powders were sintered at 500 °C with 5 °C/min heating. ZnO was synthesized through a similar process without Y^3+^.

### 2.3. Fabrication of PVDF/Y−ZnO and PVDF/ZnO Porous Electrospun Film

A total of 10 mL DMF was used to disperse 5 wt%, 10 wt%, 15 wt%, or 20 wt% Y−ZnO (or ZnO) and one hour’s sonification was used for even dispersion of fillers. Next, 1.2 g PVDF powder was dissolved into the solution with stirring and heating. Then, the solution was poured into a syringe of 10 mL. The electrospinning apparatus was settled in a chamber with humidity of ~30%, and a temperature of ~30 °C. During electrospinning, a positive voltage of 15 kV was imposed on the syringe needle, and a negative voltage of −2 kV was applied on the collector. The feed speed was 0.1 mm/min. The type of needle was 20 G, electrospun films were collected by drum collector with a rotating speed of 50 rpm on Al foil., and electrospinning distance between needle and collector was 13.5 cm. Finally, these films were dried in a vacuum at 80 °C for 24 h. The composites were labeled as n Y−Z and n Z (*n* = 5, 10, 15, or 20).

### 2.4. Fabrication of PENGs

These porous electrospun films without electrodes were corona−poled with a voltage of 20 kV and a distance of 4 cm for 1 h. Then, they were divided into several pieces with an area of 2 × 2 cm^2^. Copper foils were used as electrodes and charges were conducted by copper tape to electrometer KEITHLEY 6514. The films with copper electrodes were encapsulated by PI tapes. Finally, two stainless steel sheets were pasted outsides the device for eliminating triboelectric effect.

### 2.5. Characterization

Attenuated Total Reflection−Flourier transformed Infrared Spectroscopy (ATR−FTIR) spectra by Perkin Elmer spectrum Ⅱ (Perkin–Elmer, Waltham, USA) can quantitatively calculate the fraction of crystal phase of PVDF. X−ray diffraction (XRD) patterns by a D8 Advance from Bruker (Billerica, MA, USA) can qualitatively estimate the fraction of crystal phase of PVDF. Field emission scanning electron microscope (FESEM) images by JSM−7500F of JEOL (Tokyo, Japan) can reveal composites’ morphologies and filler dispersion. Differential scanning calorimetry (DSC) curves by TA DSC 25 (California, USA) can uncover melting enthalpies and melting points. An electrometer KEITHLEY 6514 (Tektronix, USA) can disclose the piezoelectric output performance of PENGs.

## 3. Result and Discussion

### 3.1. Morphology, Molecular Structure, and Piezoelectric Performance of PVDF Porous Electrospun Films

**Figure 1:**
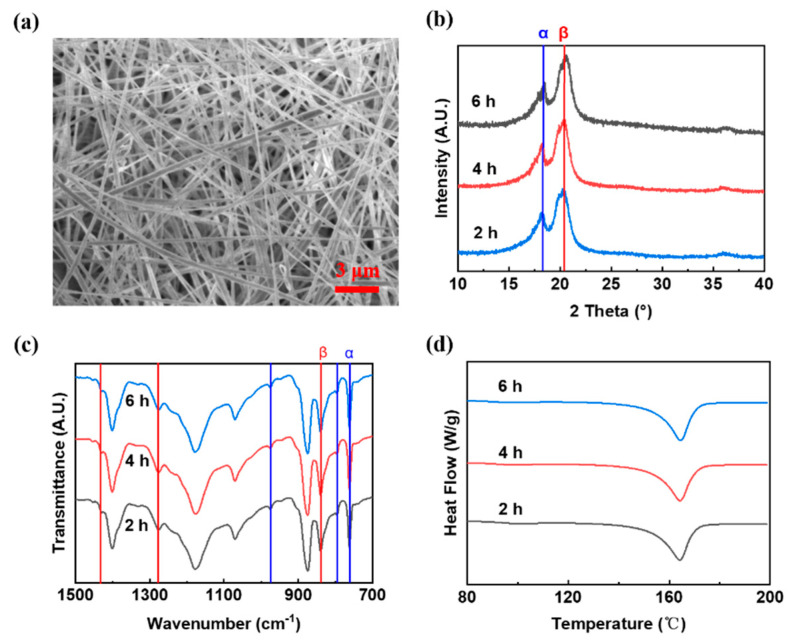
(**a**) Morphology, (**b**) XRD patterns, (**c**) FTIR spectra, and (**d**) DSC curves of PVDF porous electrospun film in different time (2 h, 4 h, 6 h).

From Figure 1, PVDF porous electrospun films was obtained through slow collector of 50 rpm during electrospinning. Our previous PVDF aligned electrospun films have reported 69% content of β phase, a crystallinity of 42%, and an average diameter of 300 nm [9]. In contrast, these PVDF porous electrospun films have a lower content of β phase (53%), lower crystallinity (~40%), and a larger average diameter of fibers (~360 nm). With increasing electrospinning time from 2 h to 6 h, the content of β phase and crystallinity almost remained constant, as in Figure 1, illustrating the negligible effect of electrospinning duration on β phase and crystallinity from FTIR spectra and DSC curves in this study. The distinction between aligned electrospun films and porous electrospun films lay less in the extent of stretching and with a lower collector speed. Gomes et al. [15] demonstrated β phase was diminished with decreasing stretching ratio and Ting et al. [16] certified that crystallinity was impaired and thickness was increased with a decreasing degree of stretching. Thus, the inferior property of porous electrospun films is ascribed to a lesser degree of stretching of fibers with a slower collector.

**Figure 2:**
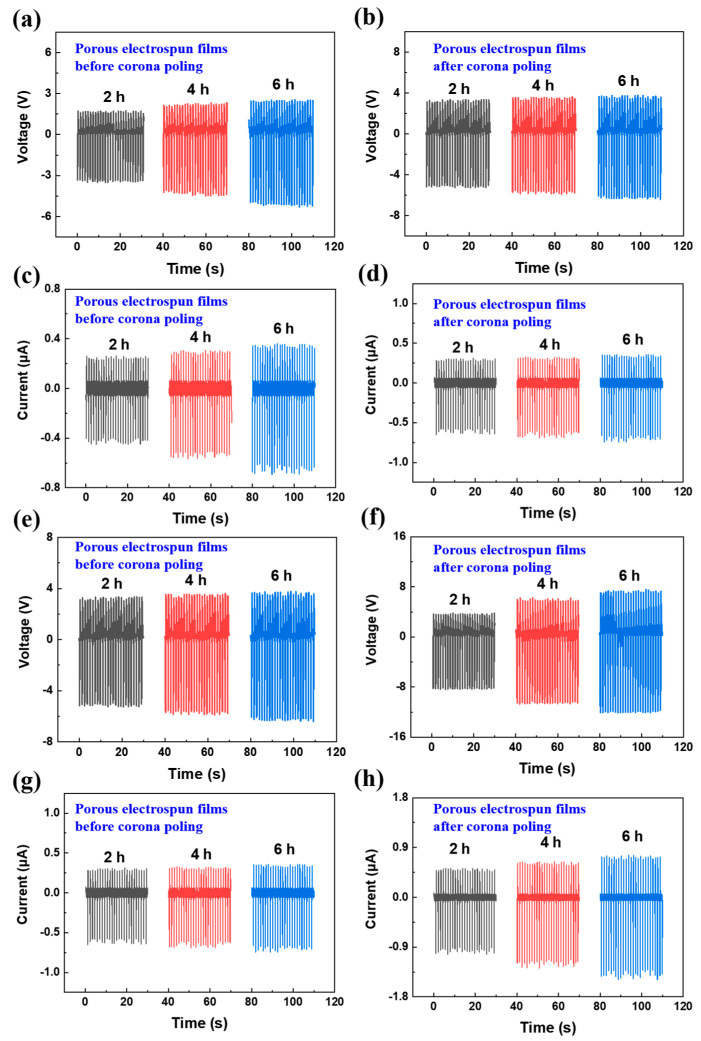
Output performance of PVDF porous electrospun films before (**a**,**c**,**e**,**g**) or after (**b**,**d**,**f**,**h**) corona−poling.

From Figure 2, with an electrospinning duration of 2 h, the output voltage and output current of PVDF porous electrospun films (2.1 V and 0.23 μA) is worse than that of PVDF aligned electrospun films (3.3 V and 0.4 μA), attributed to lower content of β−PVDF [17]. With increasing electrospinning duration, piezoelectric output of PVDF porous electrospun films are still lower than that of PVDF aligned electrospun films (Figure 2) owing to the unenhanced content of β−PVDF (Figure 1). Nonetheless, for PVDF porous electrospun films, when the electrospinning duration increased from 2 h to 6 h, the output voltage increased by ~66% (from 2.1 V to 3.5 V) and output current increased by 52% (from 0.23 μA to 0.35 μA) due to thicker porous electrospun films [18] and increasing piezoactive material [19]. Further, prolonged electrospinning duration cannot be implemented owing to consequent uncollectable fibers with electrostatic repulsion between fibers [20]. Thus, piezoelectric output performance of porous electrospun films is inferior to that of aligned electrospun films, but appropriate lengthening and electrospinning duration can improve piezoelectricity owing to increasing piezoactive material.

When the electrospinning duration increased from 2 h to 6 h, corona−poled PVDF porous electrospun films generated increasing output voltage from 8 V to 11.6 V and increasing output current from 0.91 μA to 1.3 μA, while corona−poled PVDF aligned electrospun films generated lower output voltage (4.9 V to 6 V) and lower output current (0.57 μA to 0.68 μA). Piezoelectric output of PVDF porous electrospun films surpassed that of PVDF aligned electrospun films after corona−poling, being opposite to the trends before corona−poling. Less content of β phase but more pores in PVDF porous electrospun films draw attention. Enhanced piezoelectric performance by corona poling was impossible attributed to less β phase but root from more pores.

**Figure 3:**
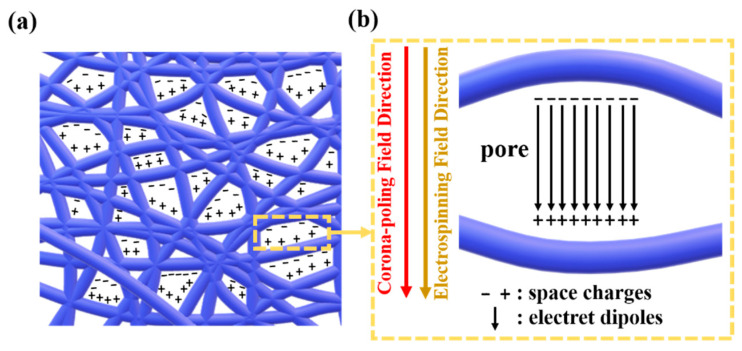
(**a**) Electret dipoles in corona−poled porous PVDF electrospun films, and (**b**) polarization effect of electrospinning field and corona−poling field on electret dipoles.

During electrospinning, pores in PVDF porous electrospun films easily form electret dipoles, which is beneficial for piezoelectricity [21]. The electrospinning process adopted an electric potential difference of 17 kV and a distance of 13.5 cm, while corona poling process promoted electric potential difference to 20 kV and shortened distance to 4 cm. The electric field improved by 297% and air in pores was polarized more effectively. During corona poling, corona ion on the surface of electrets can induce surface potential, charges can be captured by deep traps or shallow traps with adequate inner electric field, and air in pores cannot be broken down without too high of an inner electric field [22]. The inner surface of pores can produce electret dipoles, charges on the upper and lower inner surface of pores have the same quantity of charges but the opposite polarity, and electret dipoles are oriented along the direction of thickness [23]. The density of electret dipoles determines the effective polarization, the piezoelectric coefficient is positively proportional to the effective polarization, thus the piezoelectric output performance increases with an increasing number and volume of pores [24]. For comparation, aligned electrospun films and porous electrospun films were both corona−poled by a voltage of 20 kV and a distance of 4 cm for 1 h. The increasing output performance of corona−poled PVDF porous electrospun films is attributed to more electret dipoles with synergistic effect of an electrospinning electric field and a corona poling electric field, just as in Figure 3.

### 3.2. Morphology, Molecular Structure, and Piezoelectric Performance of PVDF/Y−ZnO Porous Electrospun Films

**Figure 4:**
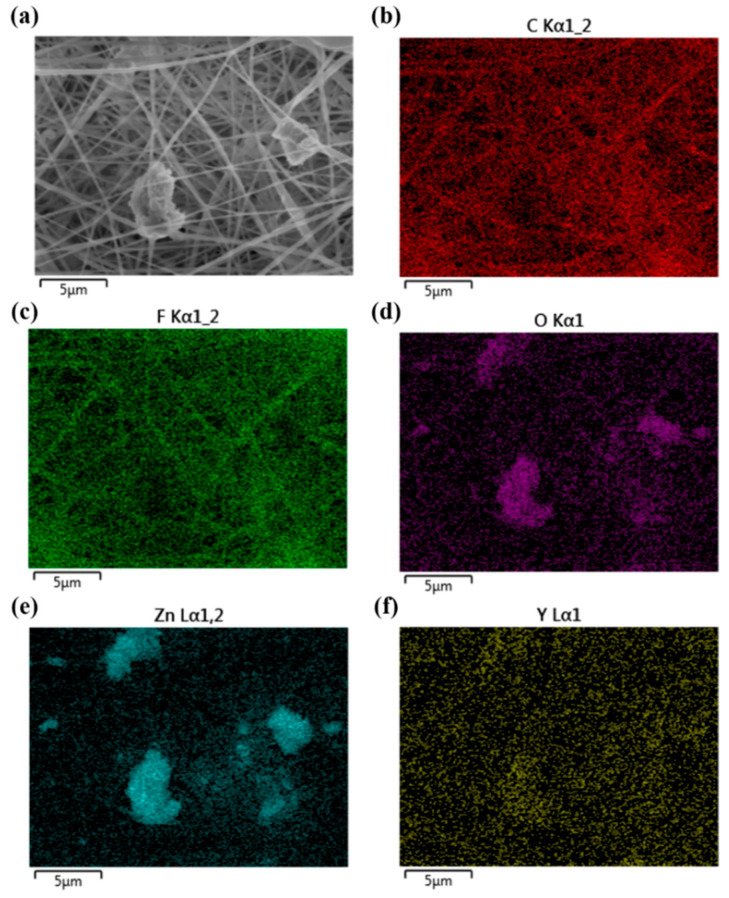
Morphology (**a**) and EDS mapping (**b**–**f**) of PVDF/Y−ZnO porous electrospun films.

**Figure 5:**
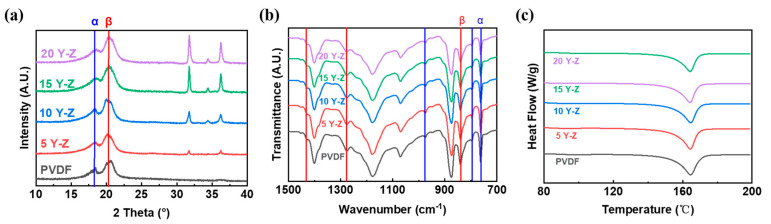
(**a**) ATR−FTIR spectra, (**b**) DSC curves, and (**c**) XRD patterns of PVDF/Y−ZnO porous electrospun films.

The merit of sheet−like Y−doped ZnO is its higher piezoelectric constant (*d*_33_* of ~420 pm/V) due to a larger polar crystal face than rod−like ZnO, according to our previous work [9]. From Figure 4, well−dispersed PVDF/Y−ZnO porous electrospun films can be achieved by electrospinning, and the polar face of Y−ZnO is parallel to the plan of electrospun films. In this case, the direction of Y−ZnO dipole deformation was consistent with that of compression, which can motivate higher piezoelectric output [25]. From Figure 5, peaks at 1275 cm^−1^ and 840 cm^−1^ correspond to β phase in electroactive PVDF phases and peak at 760 cm^−1^ stands for α−PVDF, so the content of β−PVDF (*F*_β_) can be calculated by the equation of

*F*_β_ = *A*_β_/(1.26 *A*_α_ + *A*_β_) (1)

where *A*_α_ means the absorbance of α phase and *A*_β_ means the absorbance of β phase [26]. When content of Y−ZnO increased from 0 wt% to 15 wt%, *F*_β_ increased from 53% to 60%, which can be also attested by stronger peak intensity at 20.6° from XRD patterns (Figure 5b). From Figure 5c, the crystallinity (*X*_c_) of PVDF/Y−ZnO porous electrospun films is approximate to 40%, according to the equation of

*X*_c_ = ∆*H*_m_/((1 − *ø*) ∆*H*_100%_) (2)

where ∆*H*_m_ is melting enthalpy and *ø* is fraction of filler [27].

**Figure 6:**
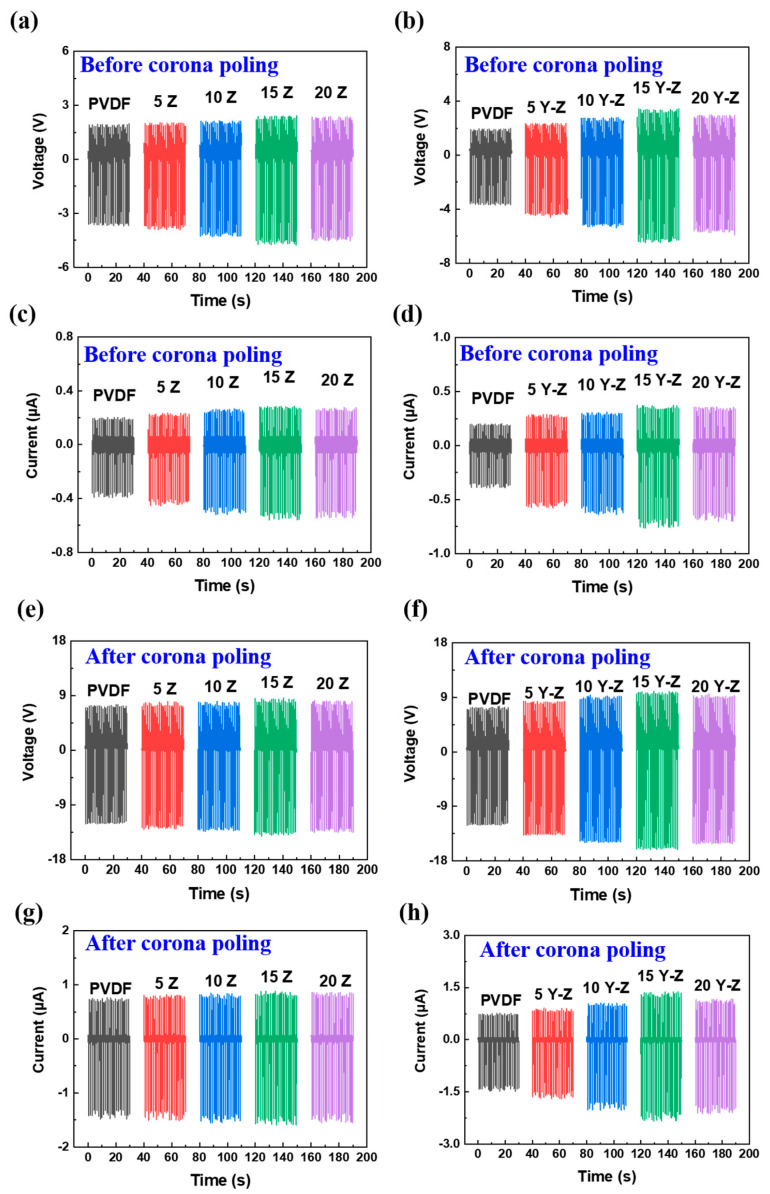
Output voltage and output current of porous PVDF/ZnO (**a**,**c**,**e**,**g**) and PVDF/Y−ZnO (**b**,**d**,**f**,**h**) electrospun films before or after corona−poling.

From Figure 6, output voltage and output current of PVDF/Y−ZnO porous electrospun films all exceeded that of PVDF/ZnO porous electrospun films either before or after corona−poling since sheet−like Y−ZnO have a larger polar crystal face with a higher piezoelectricity [8]. When content of Y−ZnO increased from 0 wt% to 15 wt%, corona−poled PVDF/Y−ZnO porous electrospun films generated an output voltage from 11.6 V to 15.3 V and an output current from 1.3 μA to 2.1 μA. Corona−poling can lead to increasing dipoles and increasing capability of charge retention, which is conducive to a piezoelectric response [28]. For electrospun films, oriented dipoles bring about polar charges on the surface of fibers, enormous local space charges form charged voids, and electret dipoles between fibers and heterogeneous surface charges can be affected by fillers with an occurrence of heterogeneous polarization and charge separation [29].

The process of piezoelectric output generation was unveiled by curves in Figure 6. Once PENGs were compressed, deformed dipoles gave rise to enhanced piezoelectric output from zero to maximum negative voltage. Upon the release of PENGs, recovered dipole led to opposite electrical signal, from negative voltage to zero and eventually to maximum positive voltage. After PENGs recovered to the original state without deformation, the remaining positive voltage equilibrated to zero. Thus, dipole deformation brought about piezoelectric output, and increased dipole or dipole alignment resulted in intensified piezoelectric output.

**Figure 7:**
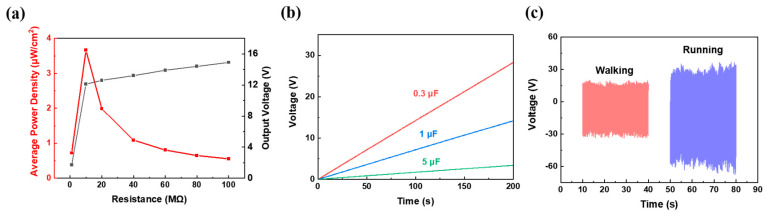
Piezoelectric output performance of PVDF/15 wt% Y−ZnO porous electrospun films: (**a**) power density, (**b**) charging curves for different capacitors, and (**c**) output voltage with people walking and running.

**Figure 8:**
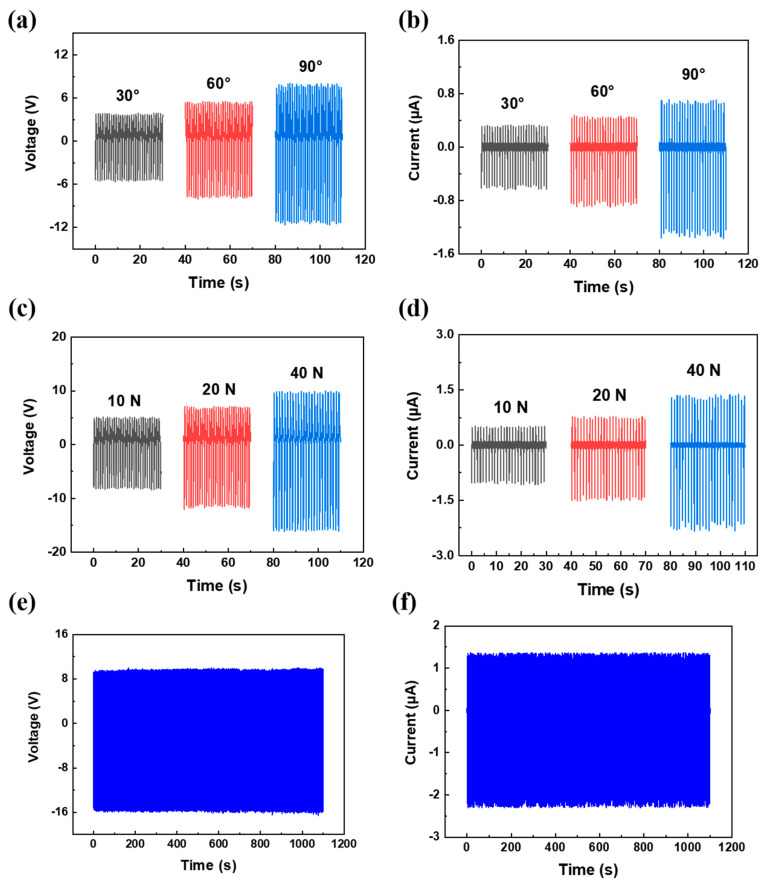
Piezoelectric output performance of porous PVDF/15 wt% Y−ZnO porous electrospun film: output voltage (**a**) and output current (**b**) with different angle of bending; output voltage (**c**) and output current (**d**) with different force; output voltage (**e**) and output current (**f**) of repeating trials of compressing and releasing.

From Figure 7, corona−poled PVDF/15 wt% Y−ZnO porous electrospun films generated a maximum output power density of 3.6 μW/cm^2^, with resistance of 10 MΩ and a charged capacitor of 5 μF to 2.8 V in 200 s. When people walked or ran on them, output voltage could reach to ~28 V and ~52 V with an effective area of 4 × 5 cm^2^. From Figure 8, when bent with increasing angles from 30° to 90°, they can generate an increasing output voltage from 5.3 V to 10.9 V and an increasing output current from 0.57 μA to 1.2 μA. When compressing force increased from 10 N to 40 N, their output voltage increased from 7.8 V to 15.3 V, and their output current increased from 0.96 μA to 2.1 μA. With compressing force increasing, piezoelectric output will rise as well because of increased piezoelectric deformation [30]. In 1100 s, an output voltage of 15.3 V and an output current of 2.1 μA remained. From Table 1, among all PENGs with a mechanism of electret dipole, this corona−poled PVDF/15 wt% Y−ZnO porous electrospun film is 18 times that of a PVDF/BiCl_3_ electrospun film owing to a high−piezoelectric Y−ZnO and a porous structure.

## 4. Conclusions

In this work, corona−poling and increasing the duration of electrospinning have a synergistic effect on piezoelectric output of PVDF porous electrospun films, superior to that of corona−poled PVDF aligned electrospun films owing to poled electret dipoles. The output performance of PVDF/Y−ZnO porous electrospun films transcends that of PVDF/ZnO porous electrospun films due to a larger polar face in Y−ZnO. Corona−poled PVDF/ 15 wt% Y−ZnO porous electrospun films generated a maximum power density of 3.6 μW/cm^2^, and they generated an output voltage of ~28 V or ~52 V when people walked or ran on them. Thus, corona−poled PVDF/15 wt% Y−ZnO porous electrospun films are potential alternatives for green energy harvesters.

## Figures and Tables

**Table 1:** Piezoelectric nanogenerator performance with mechanism of electret dipole.

Devices	Type	Area (cm^2^)	Force	Average Power Density	Ref.
PVDF−HFP/ MgCl_2_·6H_2_O	sponge−like	6.3	finger touch	60 nW	[24]
PVDF/ZnO	electrospun	1	1.5 N	0.032 μW/cm^2^	[31]
PVDF/PLA/ SnO_2_/MAPbI_3_	unpoled; spin−coated	0.0625	bending	~143 nW	[32]
PVDF/BiCl_3_	electrospun	2.25	150 g mass block	0.2 μW/cm^2^	[33]
PAN/ZnO	electrospun	16	8 N	1.08 μW/cm^2^	[34]
PVDF/3D−CNTs	electrospun	16	−	72 mW/cm^3^	[35]
P(VDF−TrFE)/ BTO	electrospun	6.25	700 N	~0.878 μW/cm^2^	[36]
PVDF/PANi/HNT	electrospun	5.1	14 g mass block	~0.049 μW/cm^2^	[37]
PVDF/ Y−ZnO	dense; corona−poled	4	40 N	3.6 μW/cm^2^	This work

## Data Availability

Not applicable.
